# Acceptability of COVID-19 Vaccines and Protective Behavior among Adults in Taiwan: Associations between Risk Perception and Willingness to Vaccinate against COVID-19

**DOI:** 10.3390/ijerph18115579

**Published:** 2021-05-23

**Authors:** Feng-Jen Tsai, Hsiu-Wen Yang, Chia-Ping Lin, Jeffrey Zen Liu

**Affiliations:** 1 Master’s Program in Global Health and Development, College of Public Health, Taipei Medical University, Taipei 110, Taiwan; spadeh2294@tmu.edu.tw; 2 Program in Global Health and Health Security, College of Public Health, Taipei Medical University, Taipei 110, Taiwan; ula0442@gmail.com (H.-W.Y.); d537108008@tmu.edu.tw (J.Z.L.)

**Keywords:** vaccine, COVID-19, risk perception, willingness, Taiwan

## Abstract

This study aims to evaluate acceptance of COVID-19 vaccines and the impact of risk perception on vaccine acceptance and personal health protective behaviors in Taiwan. A nationwide cross-sectional study was conducted from 19 to 30 October 2020; 1020 participants were included in the final analysis; chi-square and logistic regression analyses were conducted. In total, 52.7% of participants were willing to receive COVID-19 vaccines, 63.5% perceived the severity of COVID-19 in Taiwan as “not serious”, and nearly 40% were worried about COVID-19 infection. Participants with higher perceived severity of COVID-19 had significantly higher odds of refusing the vaccine (OR = 1.546), while those worried about infection had lower odds of poor health protective behaviors (OR = 0.685). Vaccine refusal reasons included “the EUA process is not strict enough” (48.7%) and “side effects” (30.3%). Those who had previously refused other vaccinations were 2.44 times more likely to refuse the COVID-19 vaccines. Participants’ age had an influence on COVID-19 vaccine acceptance. In general, the Taiwanese public’s acceptance of the vaccine was lower than that in other high-income countries. Elderly participants and those with college-level education and above who had previously refused vaccines had lower willingness to receive a COVID-19 vaccine. Risk perception was positively associated with personal health protective behaviors but negatively associated with COVID-19 vaccine acceptance.

## 1. Introduction

The World Health Organization (WHO) declared the coronavirus disease 2019 (COVID-19) a Public Health Emergency of International Concern (PHEIC) on 30 January 2020 [1]. As of January 2021, there were more than 110 million cases and 2.4 million deaths worldwide due to COVID-19 [2]. Compared with other countries, Taiwan was not seriously affected by the COVID-19 pandemic in 2020. On 21 January 2020, Taiwan reported its first confirmed imported case of 2019-nCoV, leading to the Taiwan Centers for Disease Control (TCDC) establishing the Central Epidemic Command Center (CECC) with inter-departmental horizontal coordination involving the Ministries of the Interior, Education, Transportation and Communications, etc. [3,4].

Based on its previous experience from the SARS outbreak, the Taiwanese government took prompt and effective action to mitigate and control COVID-19 within the country. As of May 2021, Taiwan has had 1145 confirmed cases and 12 deaths, with a majority of cases being imported [5]. This controlled outbreak has led to Taiwan being listed as one of the countries with moderate economic impact as its gross domestic product (GDP) declined by less than 1% [6].

Taiwan has effectively mitigated the effects of COVID-19 through border management, health inspections, contact tracing, and other public health measures [7]. However, vaccines are still listed as one of the prioritized strategies for achieving herd immunity for further policy movement [8]. Taiwan has purchased a large number of vaccines and aims to begin a large-scale vaccination program in the following months [9]. According to the WHO Strategic Expert Advisory Group (SAGE) and the Global Vaccine Safety Advisory Committee (GACVS), one of the goals of the COVID-19 vaccine communication plan is to achieve high acceptance and uptake [10]. The current acceptance of COVID-19 vaccines globally is approximately 35% to 98%, which varies greatly from country to country, further indicating the need for policymakers to understand public perceptions before large-scale vaccination [11,12,13]. This study was conducted to fill the information gap regarding this issue in Taiwan.

The perception of the severity of the COVID-19 pandemic is related to people’s awareness of risks and encourages the public to further participate in disease prevention measures [14,15]. This can also be verified as it is related to vaccinations during the H1N1 influenza pandemic and the subsequent formulation of prevention policies, corresponding to the theoretical structure of threat and response assessments in the use of the individual healthy behavior models [16,17,18]. When the public’s perception of the severity of COVID-19 is higher, the willingness to implement preventative measures is higher [19,20,21]. Previous studies based on this theory showed that perceived severity of COVID-19 and the fear of contracting COVID-19 were positively associated with vaccine acceptance [22]. In addition, higher levels of risk exposure and negative attitudes toward general vaccination were associated with low vaccine acceptance [23]. Since the COVID-19 pandemic is not severe in Taiwan, the authors wondered about whether the impact of risk perception on vaccine acceptance is still sustained in Taiwan. Therefore, we conducted this national survey to evaluate the impact of risk perception on public acceptance regarding COVID-19 vaccines and the factors influencing willingness to vaccinate in Taiwan. In addition, we investigated the impact of risk perception on recommended personal health protective behavior, such as handwashing, mask wearing, and social distancing. Although vaccines and practicing personal health protective behaviors are measures recommended by the government, the decision-making process for vaccination is complicated and potentially affected by a variety of factors such as trust in the government, historical influences, and religion [24,25]. This study looks into the impact of risk perception on practicing personal health protective behavior and how it differs from its impact on vaccination acceptance.

Therefore, the aims of this study are to (1) understand public acceptance of COVID-19 vaccines in Taiwan and influencing factors; (2) evaluate the association between risk perception and public acceptance of COVID-19 vaccines; and (3) evaluate the association between risk perception and personal health protective behaviors.

## 2. Materials and Methods

### 2.1. Research Design

The cross-sectional study was conducted from 19 to 30 October 2020. Participants were adults aged over 20, fluent in Mandarin and/or Taiwanese, who lived in Taiwan.

Data were collected by an academic institution’s public opinion polling center through an anonymous national telephone survey with structured questionnaire interviews. Stratified sampling was used during the survey period. A total of 14,866 random telephone calls to respondents from different counties and cities across the nation were made, with 1077 respondents completing the survey—a response rate of 7.24%. Those who responded to the telephone survey did not receive any incentive. Further weighting was applied in order to maintain full representation of the country’s population. Participants’ individual characteristics, including age, gender, education, religion, and work status were also collected. In total, 1020 participants were included in the final analysis after excluding participants who did not respond regarding their willingness to receive a COVID-19 vaccine. The analytic sample was not different from the 1077 participants who took the survey.

### 2.2. Measures

The research team developed the questionnaire to examine risk perception and willingness to vaccinate against COVID-19 among Taiwan’s population based on a 2014 national survey [26]. The questionnaire was then reviewed by a panel of experts with backgrounds in anthropology, immunology, law, medicine, public health, and sociology. Revisions were made based on the expert panel’s comments. The revised questionnaire was further pilot-tested with 15 adults.

The questionnaire used in this research includes six dimensions: “sources of vaccine information”, “factors affecting willingness to vaccinate”, “attitude toward different vaccine resource allocation ethical approach”, and “risk perception, willingness to vaccinate, and health behaviors regarding COVID-19 in Taiwan”. This study specifically analyzed the parts of “factors affecting willingness to vaccinate” and “risk perception, willingness to vaccinate, and personal health protective behaviors regarding COVID-19 in Taiwan”.

#### 2.2.1. Risk Perception, Vaccine Knowledge, and Previous Experience with Vaccination

Participants’ risk perception regarding COVID-19 in Taiwan was evaluated using two questions: “What do you think of the severity of the COVID-19 outbreak in Taiwan?” and “To what level do you worry about getting COVID-19?” Responses were based on a 5-point Likert scale, ranging from “very serious” to “not very serious” and from “very worried” to “not very worried”.

Participants’ perceptions of their knowledge regarding vaccines were assessed using a self-evaluated question: “Please rate your understanding regarding vaccines from 1 (lowest) to 10 (highest)”. Participants’ previous experience with vaccinations was assessed with the following question: “Have you ever refused a vaccination while it is recommended by the government for yourself and your kids?”

#### 2.2.2. Willingness to Vaccinate and Personal Health Protective Behaviors Regarding COVID-19

Participants’ vaccine acceptance was assessed using the following question: “Are you willing to get a COVID-19 vaccine if the vaccine is provided through the Emergency Use Authorization (EUA) process for approval?” Responses included “I am willing to get the COVID 19 vaccine” and “I am not willing to get the COVID 19 vaccine”; their reasons as to why they were not willing to receive a COVID-19 vaccine were collected for further analysis.

Participants’ personal health protective behaviors were assessed with 3 questions: “Do you agree with the statement: ‘I wash my hands more frequently compared to before the COVID-19 pandemic’”; “Do you agree with the statement: ‘I wear masks more frequently compared to before the COVID-19 pandemic’”, and “In the past 30 days, how carefully have you maintained social distancing (1.5 m indoors and 1-m outdoors)?” Responses were based on a 5-point Likert scale, ranging from “strongly agree” to “strongly disagree” and from “always” to “never”.

### 2.3. Statistical Analysis

A Chi-square test was used to compare individual characteristics, including sex, age, education, religion, occupational status, and willingness to vaccinate against COVID-19, as well as “self-rated vaccine knowledge”, “experience of vaccine refusal”, “severity of the COVID-19 pandemic in Taiwan”, and “concern about contracting COVID-19” between participants willing and unwilling to receive a COVID-19 vaccine.

The 5-point Likert scale of the three personal health protective behaviors (handwashing, masks wearing, and keeping social distancing) was summarized as the overall indicator of “Health Protective Behavior”, which was further classified for further analysis into the “Good behavior” and “Poor behavior” groups via the median score. The health protective behavior data skewed left. There are two reasons to combine all “health protective behaviors” into one single measure. First, all three health protective behaviors were recommended by the Taiwan CDC, and adopting all three health protective behaviors could protect individuals from the risk of contracting COVID-19. Second, participants’ responses regarding behaviors such as handwashing and wearing masks were comparatively high, so we combined them to achieve better grouping of participants into good and poor health behavior groups.

Participants’ perceived knowledge of vaccines was categorized into three groups—“low”, “medium”, and “high”—for analysis in our study due to concerns that the data were not normally distributed.

Regression analysis was then applied to determine the correlation between willingness to receive a COVID-19 vaccine and risk perception. Bivariate analysis was initially used to understand the relationship between willingness to receive COVID-19 vaccination and potential confounding factors including age, sex, education, religion, occupational status, self-rated vaccine knowledge, and experience of vaccine refusal. Logistic regression was then applied to determine the statistical significance of the association between participants’ willingness to receive COVID-19 vaccination and their risk perception. This approach was also applied to understand the association between participants’ health protective behaviors and their risk perception. Our study’s dependent variables are “unwillingness to take COVID-19 vaccination” and” poor health protective behaviors in COVID-19 prevention”, while the independent variables are “severity of COVID-19 pandemic in Taiwan” and “concern regarding COVID-19 infection”. Individual characteristics including sex, education, age, occupational status, and experience of vaccine refusal are covariates of interest.

The odds ratios (ORs) and 95% confidence intervals (CIs) were also calculated, and the significance level was set at 0.05. All statistical analyses were conducted in SPSS version 18 (IBM, Armonk, NY, USA).

## 3. Results

### 3.1. Individual Characteristics of Participants

The individual characteristics of participants are provided in Table 1. Among 1020 participants, the majority were women (56.7%), and the age group with the highest proportion (43.7%) were 40–59 years old. Coupled with participants above 60 years old, the proportion of participants over 40 years old in the entire survey was 77.3%. In total, 54.8% of participants had undergraduate degrees or higher, and 61.3% of them were currently employed. Approximately one third of participants stated that they had no religious beliefs (36.0%).

Regarding willingness to receive a COVID 19 vaccine, 52.7% of participants were willing to receive the vaccine, while nearly half (47.3%) were unwilling to receive it. The main reason for refusing the vaccine was concern that “the EUA process is not strict enough” (296/608, 48.7%), followed by concern regarding “side effects” (184/608, 30.3%). All of the options and responses regarding participants’ vaccine refusal are provided in the Supplementary Materials.

Chi-square analysis showed that sex, education, and occupational status were all significantly different between the “willing and unwilling to take COVID-19 vaccine” groups. There were significantly more female participants in the unwilling group. The percentage of participants with undergraduate degrees or higher and the percentage of participants that were currently employed were both significantly higher among participants who were not willing to receive a COVID-19 vaccine.

Within each individual factor, the percentage of participants unwilling to receive a COVID-19 vaccine is higher among women (50.17%) than among men (43.44%) and higher among those who were employed (49.76%) than those unemployed/retired (43.08%). Regarding religion, the percentage of participants unwilling to receive a COVID-19 vaccine is highest among Christians (52.83%), but the difference is not significant.

### 3.2. Comparison of Risk Perception and Factors Influencing Vaccination Willingness between Willing and Unwilling to Receive COVID-19 Vaccine Group

Comparisons of risk perception, knowledge, and experience of vaccine refusal between the willing and unwilling to receive a COVID-19 vaccine groups are shown in Table 2. Overall, more than 70% of participants self-rated their perception of their knowledge regarding vaccines as medium to high, and 28.6% of them have previously refused vaccinations for themselves. Regarding risk perception, around 63.5% of participants perceived the spread of the COVID-19 pandemic in Taiwan as “not serious”, and nearly 40% responded that they were “not worried” about COVID-19 infection.

Participants’ experience with refusing previous vaccines and their perception of the severity of the COVID-19 pandemic in Taiwan were both significantly different between the willing and unwilling to receive a COVID-19 vaccine groups. The percentage of participants with experience of vaccine refusal was significantly higher in the unwilling group (39.3% in the unwilling group vs. 19.1% in the willing group), but the percentage of participants who perceived the COVID-19 pandemic in Taiwan as serious was also significantly higher in the unwilling group (17.1% in the unwilling group vs. 11.6% in the willing group).

### 3.3. Association between Participants’ Willingness to Receive COVID-19 Vaccine and Their Risk Perception

Logistic regression analysis showed that participants’ sex, education, age, occupational status, experience of vaccine refusal, and perceived severity of the COVID-19 pandemic in Taiwan were significantly associated with their willingness to receive COVID-19 vaccination (Table 3). Participants who were women, had higher education levels, and were currently employed were more unwilling to receive a COVID 19 vaccine. In addition, participants aged 60 and above had ORs of 1.967 for unwillingness to receive a COVID-19 vaccine, while participants aged between 40 to 59 years old also had significantly higher ORs of unwillingness to receive a COVID-19 vaccine than participants aged less than 40 years old.

Participants who perceived the COVID-19 pandemic in Taiwan as “serious” had significantly higher odds of unwillingness to receive a COVID-19 vaccine. Participants who had previously refused vaccinations had ORs of 2.435 for refusal to receive a COVID-19 vaccine, compared to those who had never refused to receive vaccines.

### 3.4. Association between Participants’ Health Protective Behaviors and Their Risk Perception

More than 90% of participants increased their frequency of handwashing and mask wearing behaviors in the previous month, and more than 70% of participants practiced social distancing to prevent COVID-19 infection.

The association between participants’ health protective behaviors and their risk perception is shown in Table 4. From the analysis, participants’ sex and the status of feeling worried about contracting COVID-19 were significantly associated with personal health protective behaviors.

Female participants had fewer odds of not adopting health protective behaviors than male participants (OR = 0.737), and participants who worried about COVID-19 infection had lower odds of poor health protective behaviors (OR = 0.685).

In addition, further analysis showed that there were more participants with poor health protective behaviors in the group of participants unwilling to receive a COVID-19 vaccine (data not shown).

## 4. Discussion

This is the first study aimed at evaluating the acceptance of COVID-19 vaccines in Taiwan. Furthermore, this study was used to determine the impact of risk perception on both vaccine acceptance and health protective behaviors. Nearly half of the participants were unwilling to receive a COVID-19 vaccine (willing vs. unwilling: 52.7% vs. 47.3%). Participants who were worried about COVID-19 infection were more willing to adopt health protective behaviors, but participants who perceived the risk of the COVID-19 pandemic in Taiwan to be serious were less willing to receive a COVID-19 vaccine. Moreover, willingness to accept the new vaccine is affected by past experiences, as participants who had previously refused vaccines were more likely to be unwilling to receive a COVID-19 vaccine.

Taiwan’s acceptance rate of COVID-19 vaccines is lower than those of most countries around the world and lower than those of France and Sweden in particular, which already have low acceptance rates. This phenomenon might be due to the low number of cases of COVID-19 in Taiwan. The fact that vaccine coverage rates are generally over 90% in Taiwan further indicates the impact of distrust of the government as a potential explanation for our findings, similar to other countries. The main reasons that participants were unwilling to receive a COVID-19 vaccine were “the EUA (Emergency Use Authorization) process is not strict enough” and “side effects” of the vaccine, displaying a distrust of governmental control of vaccine safety and quality through the EUA process.

Surprisingly, the study results showed that participants who perceived the risk of the COVID-19 pandemic in Taiwan to be serious were less willing to receive a COVID-19 vaccine. The possible explanation for this unexpected phenomenon might be the participants’ sensitivity toward these issues. Participants who perceived the risk of the COVID-19 pandemic in Taiwan to be “serious” might be individuals who are more sensitive to health-related issues. These participants may be more hesitant to accept the vaccine since the COVID-19 vaccines available are comparatively new, with high uncertainty in regard to possible side effects. Further studies are needed to understand this phenomenon.

According to the WHO, people over 60 years old are at high risk of severe COVID-19 infection [27]. Although the WHO SAGE did not consider age to be a priority for vaccine distribution, it declared that vulnerability to COVID-19 should be considered for vaccine allocation [28]. In previous studies, willingness to receive vaccines in high-risk groups, such as older adults, patients with chronic respiratory diseases, and frontline medical personnel, was found to be relatively high [29,30]. However, in our survey, participants aged 60 years and above had ORs of 2.00 of unwillingness to receive a COVID-19 vaccine, and participants aged between 40 and 59 years also had significantly higher ORs for unwillingness to receive a COVID-19 vaccine compared to participants aged less than 40 years. This finding indicates the need to further understand the practical considerations of this age group through in-depth interview research in the future.

In addition, we found that the impact of sociodemographic backgrounds in Taiwan was different compared to that of other countries. Previous studies in other countries showed that populations with higher education levels had higher willingness to receive the vaccine; however, this study’s findings identified the reverse pattern [31,32,33,34,35]. This might be due to distrust of the government by participants with higher education levels, indicating the need for the government to develop effective communication strategies for COVID-19 vaccination for the population.

Participants who worried about COVID-19 infection were more willing to adopt health protective behaviors, but participants who perceived the risk of the COVID-19 pandemic in Taiwan to be serious were less willing to receive a COVID-19 vaccine. This indicates the different aspects that participants must consider when adopting personal health protective measures against unknown diseases such as COVID-19. Participants with a higher risk perception of COVID-19 adopted more non-invasive personal health protective behaviors, such as handwashing, mask wearing, and social distancing, but showed less willingness to receive a COVID-19 vaccine. One of the possible explanations of this phenomenon might be that partaking in non-invasive health protective behaviors only mildly inconveniences the user, unlike the potentially serious side effects of vaccination. Therefore, the impact of risk perception on vaccine acceptance and health protective behaviors is different in our study. The research results echoed the findings and argument that “the public trust in the government is the key to vaccine acceptance” [12,36,37]. Further studies are needed to understand the reason for this phenomenon.

### Limitations

This study had several limitations. First, due to the use of home telephone interviews from 6.00 p.m. to 10.00 p.m. on weekdays, our participants were mainly in the middle-aged group and above. Second, the investigation period was the month of October 2020, when there was less information regarding COVID-19 vaccines than there is now. However, the findings still reflect the need to develop an effective communication strategy for vaccine acceptance in Taiwan. Third, the relationships can only be considered to be associations rather than causal due to the nature of cross-sectional design; we might also not be able to rule out reporting bias due to the data collection method. Fourth, it should be noted that the low response rate in the telephone survey may cause sample bias and overestimation results, and acceptance of COVID-19 vaccines in the real world may be lower.

## 5. Conclusions

Our findings indicate that Taiwan’s vaccine acceptance was relatively low and affected by previous vaccination experiences. Elderly participants, women, and those with higher education levels were less willing to receive a COVID-19 vaccine, and participants with a higher risk perception of COVID-19 adopted more non-invasive personal health protective behaviors, such as handwashing, mask wearing, and social distancing, but showed less willingness to receive a COVID-19 vaccine. We suggest that in-depth inquiries should be conducted regarding the considerations of specific ethnic groups who refuse to receive a COVID-19 vaccine, and risk communication strategies should be developed.

## Figures and Tables

**Table 1:** Basic demographic characteristics affecting COVID-19 vaccine uptake intention.

Variables	Total (*n* = 1020)		Unwilling (*n* = 482)		Willing (*n* = 538)		*p*-Value
	*n*	%	*n*	%	*n*	%	
**Sex (*n* = 1020)**							
Male	442	43.3%	192	39.8%	250	46.5%	0.033
Female	578	56.7%	290	60.2%	288	53.5%	
**Education (*n* = 1014)**							
High school and below	458	45.2%	176	36.8%	282	52.6%	<0.001
College	479	47.2%	259	54.2%	220	41.0%	
Master and above	77	7.6%	43	9.0%	34	6.3%	
**Age groups (*n* = 1020)**							
20~39	187	17.4%	83	17.2%	104	19.3%	0.238
40~59	471	43.7%	236	49.0%	235	43.7%	
Above 60 (including)	362	33.6%	163	33.8%	199	37.0%	
**Religion (*n* = 1016)**							
Folk religion (including Taoism and Yiguandao)	291	28.6%	127	26.5%	164	30.5%	0.326
Christianity	53	5.2%	28	5.8%	25	4.7%	
Buddhism	306	30.1%	141	29.4%	165	30.7%	
No religion	366	36.0%	183	38.2%	183	34.1%	
**Occupational status (*n* = 1007)**							
Employed	617	61.3%	307	64.6%	310	58.3%	0.039
Unemployed/retired	390	38.7%	168	35.4%	222	41.7%	

**Table 2:** Knowledge and experience of vaccination and risk perception variables affecting COVID-19 vaccine uptake intention.

Variables	Total (*n* = 1020)		Unwilling (*n* = 482)		Willing (*n* = 538)		*p*-Value
	*n*	%	*n*	%	*b*	%	
**Knowledge (*n* = 1020)**							
High scores (7–10)	346	33.9%	155	32.2%	191	35.5%	0.497
Medium scores (5–6)	391	38.3%	192	39.8%	199	37.0%	
Low scores (1–4)	283	27.7%	135	28.0%	148	27.5%	
**Experience of vaccine refusal (*n* = 1010)**							
Ever	289	28.6%	187	39.3%	102	19.1%	<0.001
None	721	71.4%	289	60.7%	432	80.9%	
**Severity of the COVID-19 pandemic in Taiwan (*n* = 1002)**							
Serious	142	14.2%	81	17.1%	61	11.6%	0.046
Not serious/slightly serious	224	22.4%	102	21.5%	122	23.1%	
Not serious	636	63.5%	292	61.5%	344	65.3%	
**Worried about contracting COVID-19 (*n* = 1017)**							
Worried	638	62.7%	298	61.8%	340	63.6%	0.570
Not worried	379	37.3%	184	38.2%	195	36.4%	

**Table 3:** Logistic regression model of unwillingness to receive COVID-19 vaccination.

Estimated Odds Ratios of Unwillingness to Receive COVID-19 Vaccination				
Variables	*Crude OR (95% CI)*	*p-Value*	*Adjusted OR (95% CI)*	*p-Value*
**Sex**				
Male	(ref)		(ref)	
Female	1.311 (1.022–1.682)	0.033	1.344 (1.016–1.779)	0.039
**Education**				
High school and below	(ref)		(ref)	
College	1.866 (1.454–2.447)	<0.001	2.100 (1.550–2.844)	<0.001
Master and above	2.026 (1.244–3.300)	0.005	2.399 (1.396–4.123)	0.002
**Age groups**				
20~39	(ref)		(ref)	
40~59	1.258 (0.895–1.769)	0.186	1.667 (1.145–2.427)	0.008
Above 60 (including)	1.026 (0.720–1.464)	0.886	1.967 (1.254–3.086)	0.003
**Occupational status**				
Unemployed/retired	(ref)		(ref)	
Employed	1.309 (1.014–1.689)	0.039	1.442 (1.056–1.970)	0.021
**Experience of vaccine refusal**				
None	(ref)		(ref)	
Ever	2.740 (2.064–3.639)	<0.001	2.435 (1.806–3.282)	<0.001
**Severity of the COVID-19 pandemic in Taiwan**				
Not serious	(ref)		(ref)	
Not serious/slightly serious	0.985 (0.726–1.337)	0.923	1.042 (0.751–1.445)	0.806
Serious	1.564 (1.084–2.258)	0.017	1.546 (1.031–2.320)	0.035
**Worried about contracting COVID-19**				
Not worried	(ref)		(ref)	
Worried	0.929 (0.720–1.198)	0.570	0.795 (0.601–1.053)	0.110

**Table 4:** Logistic regression model of poor health protective behaviors for COVID-19 prevention.

Estimated Odds Ratios of Poor Health Protective Behaviors for COVID-19 Prevention				
Variables	*Crude OR (95% CI)*	*p-Value*	*Adjusted OR (95% CI)*	*p-Value*
**Sex**				
Male	(ref)		(ref)	
Female	0.694 (0.540–0.892)	0.004	0.737 (0.564–0.964)	0.026
**Education**				
High school and below	(ref)		(ref)	
College	0.843 (0.652–1.091)	0.195	0.775 (0.580–1.037)	0.086
Master and above	1.023 (0.629–1.664)	0.927	0.937 (0.555–1.583)	0.809
**Age groups**				
20~39	(ref)		(ref)	
40~59	0.900 (0.640–1.266)	0.545	0.861 (0.599–1.238)	0.420
Above 60 (including)	0.872 (0.612–1.244)	0.451	0.810 (0.526–1.246)	0.337
**Occupational status**				
Unemployed/retired	(ref)		(ref)	
Employed	1.207 (0.935–1.557)	0.149	1.082 (0.806–1.466)	0.583
**Severity of the COVID-19 pandemic in Taiwan**				
Not serious	(ref)		(ref)	
Not serious/slightly serious	1.116 (0.820–1.519)	0.485	1.195 (0.870–1.641)	0.272
Serious	0.718 (0.498–1.035)	0.075	0.842 (0.573–1.238)	0.382
**Worried about contracting COVID-19**				
Not worried	(ref)		(ref)	
Worried	0.650 (0.502–0.842)	0.001	0.685 (0.522–0.900)	0.007

## Data Availability

The data presented in this study are available on request from the corresponding author.

## Supplementary Materials

The following are available online at https://www.mdpi.com/article/10.3390/ijerph18115579/s1, Table S1: Reasons for Unwillingness to Take COVID-19 Vaccination, Table S2: Reasons for Previously Refusing Vaccines.
