# Supplementary material for: Acceptability of COVID-19 Vaccines and Protective Behavior among Adults in Taiwan: Associations between Risk Perception and Willingness to Vaccinate against COVID-19
Source: Int J Environ Res Public Health. 2021 May 23;18(11):5579. doi: 10.3390/ijerph18115579 (PMC8197151; doi:10.3390/ijerph18115579)
Supplement: Supplementary file 1 [file ijerph-18-05579-s001.zip › ijerph-1166315-supplementary.pdf]

Supplementary Materials

Table S1. Reasons for Unwillingness to Take COVID-19 Vaccination.

Description	Frequency	Percentage of Reasons for Unwillingness (N = 482)	Frequency Percentage of Reasons for Unwillingness (N = 608)
Not rigorous in the fast-tracking process	296	61.41%	48.68%
Worried about side effects	184	38.17%	30.26%
No need	54	11.20%	8.88%
Uncertainty of utility	51	10.58%	8.39%
Don't know/ No comments	10	2.07%	1.64%
Priority for people in need	7	1.45%	1.15%
Fear of needles or injections	5	1.04%	0.82%
No response	1	0.21%	0.16%

Table S2. Reasons for Previously Refusing Vaccines.

Description	Frequency	Percentage of Reasons for Refusal Experience (N = 313)	Frequency Percentage of Reasons for Refusal Experience (N = 381)
Worried about side effects	116	37.06%	30.45%
No need	56	17.89%	14.70%
Feel healthy	46	14.70%	12.07%
Worried about vaccine quality	41	13.10%	10.76%
Busy or inconvenient	34	10.86%	8.92%
Poor physical condition	17	5.43%	4.46%
Don't know/ No comments	15	4.79%	3.94%
Distrust of modern medicine	13	4.15%	3.41%
Distrust the government's advice	13	4.15%	3.41%
Medical not recommended	7	2.24%	1.84%
Personal bad experience	7	2.24%	1.84%
Fear of needles or injections	6	1.92%	1.57%
It's fine if others have a vaccination	2	0.64%	0.52%
Out of stock	2	0.64%	0.52%
No response	2	0.64%	0.52%
Others	4	1.28%	1.05%
